# Supplementary material for: Learning Shapes the Energy Cost of Neural Tasks
Source: bioRxiv. 2026 Jul 2:2026.07.01.735889. Preprint. [Version 1] doi: 10.64898/2026.07.01.735889 (PMC13345034; doi:10.64898/2026.07.01.735889)
Supplement: Supplement 1 [file NIHPP2026.07.01.735889v1-supplement-1.pdf]

bioRxiv preprint doi: https://doi.org/10.64898/2026.07.01.735889; this version posted July 2, 2026. The copyright holder for this preprint (which was not certified by peer review) is the author/funder, who has granted bioRxiv a license to display the preprint in perpetuity. It is made available under a CC-BY 4.0 International license.

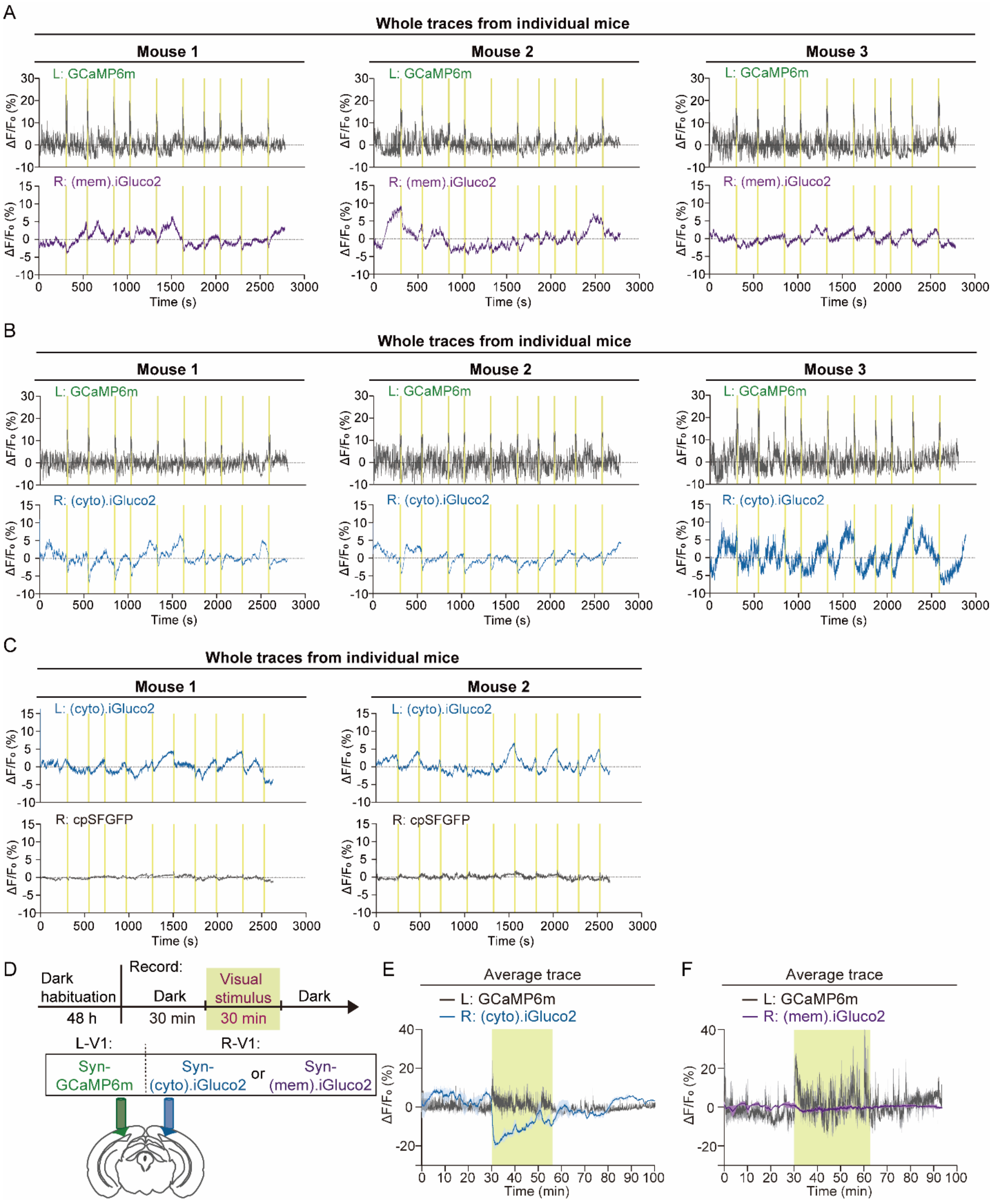

bioRxiv preprint doi: https://doi.org/10.64898/2026.07.01.735889; this version posted July 2, 2026. The copyright holder for this preprint (which was not certified by peer review) is the author/funder, who has granted bioRxiv a license to display the preprint in perpetuity. It is made available under a CC-BY 4.0 International license.

Fig. S1. Visual stimulation reduces glucose levels in primary visual cortex neurons.
(A) Individual mouse traces showing GCaMP6m and (mem).iGluco2 signals recorded from the left (L, in green) and right (R, in purple) V1 neurons of the same animal.
(B) Individual mouse traces showing GCaMP6m and (cyto).iGluco2 signals recorded from the left and right V1 neurons of the same animal.
(C) Individual mouse traces showing (cyto).iGluco2 and cpSFGFP signals recorded from the left and right V1 neurons of the same animal.
(D) Schematic showing the long-term 30-min visual stimulation paradigm, sensor injection, and fiber placement for the experiments in E and F.
(E) Average fiber photometry traces showing GCaMP6m signals in the left V1 and (cyto).iGluco2 signals in the right V1 of the same animal during visual stimulation (n = 3 mice).
(F) Average fiber photometry traces showing GCaMP6m signals in the left V1 and (mem).iGluco2 signals in the right V1 of the same animal during visual stimulation (n = 3 mice).

bioRxiv preprint doi: https://doi.org/10.64898/2026.07.01.735889; this version posted July 2, 2026. The copyright holder for this preprint (which was not certified by peer review) is the author/funder, who has granted bioRxiv a license to display the preprint in perpetuity. It is made available under a CC-BY 4.0 International license.

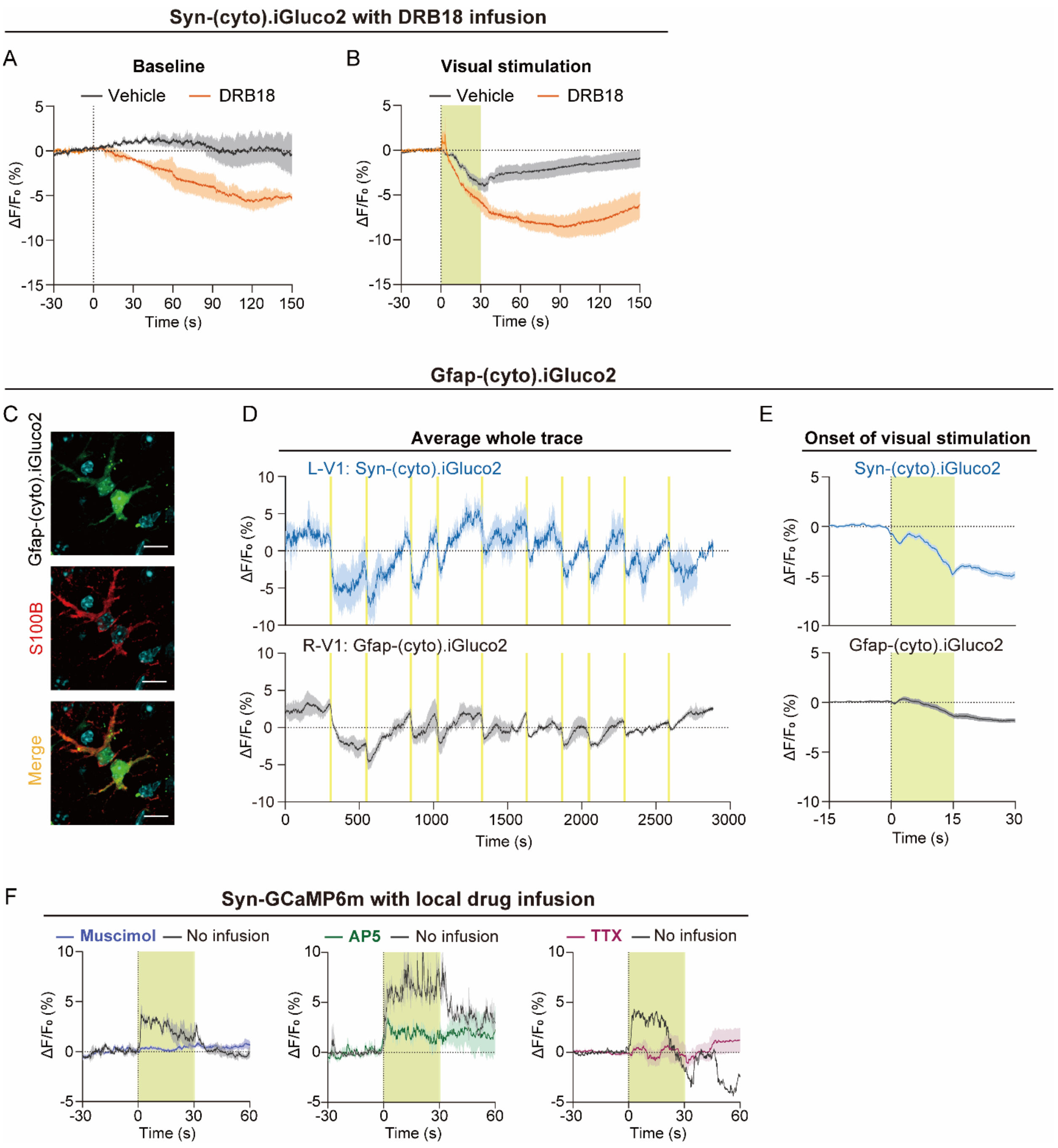

Fig. S2. Visual stimulation-induced neuronal glucose drop is driven by neuronal activity.
(A-B) (cyto).iGluco2 signals in V1 neurons following vehicle or pan-GLUT transporter inhibitor (DRB18) infusion under baseline (A) and visual stimulation (B) conditions.
(C) Histology showing colocalization of Gfap-(cyto).iGluco2 expression with the astrocyte marker S100B in V1 cortex.
(D) Average fiber photometry traces showing Syn-(cyto).iGluco2 signals in the left V1 (upper) and Gfap-(cyto).iGluco2 signals in the right V1 (lower) of the same animal during visual stimulation (n = 3 mice).

bioRxiv preprint doi: https://doi.org/10.64898/2026.07.01.735889; this version posted July 2, 2026. The copyright holder for this preprint (which was not certified by peer review) is the author/funder, who has granted bioRxiv a license to display the preprint in perpetuity. It is made available under a CC-BY 4.0 International license.

(E) Signal changes aligned to the onset of visual stimulation (n = 3 mice)

(F) GCaMP6m signal changes aligned to visual stimulation onset with and without local drug infusion. A $GABA_A$ receptor agonist (muscimol), an NMDAR antagonist (AP5), or a sodium channel blocker (TTX) was infused into V1 through the cannula.

bioRxiv preprint doi: https://doi.org/10.64898/2026.07.01.735889; this version posted July 2, 2026. The copyright holder for this preprint (which was not certified by peer review) is the author/funder, who has granted bioRxiv a license to display the preprint in perpetuity. It is made available under a CC-BY 4.0 International license.

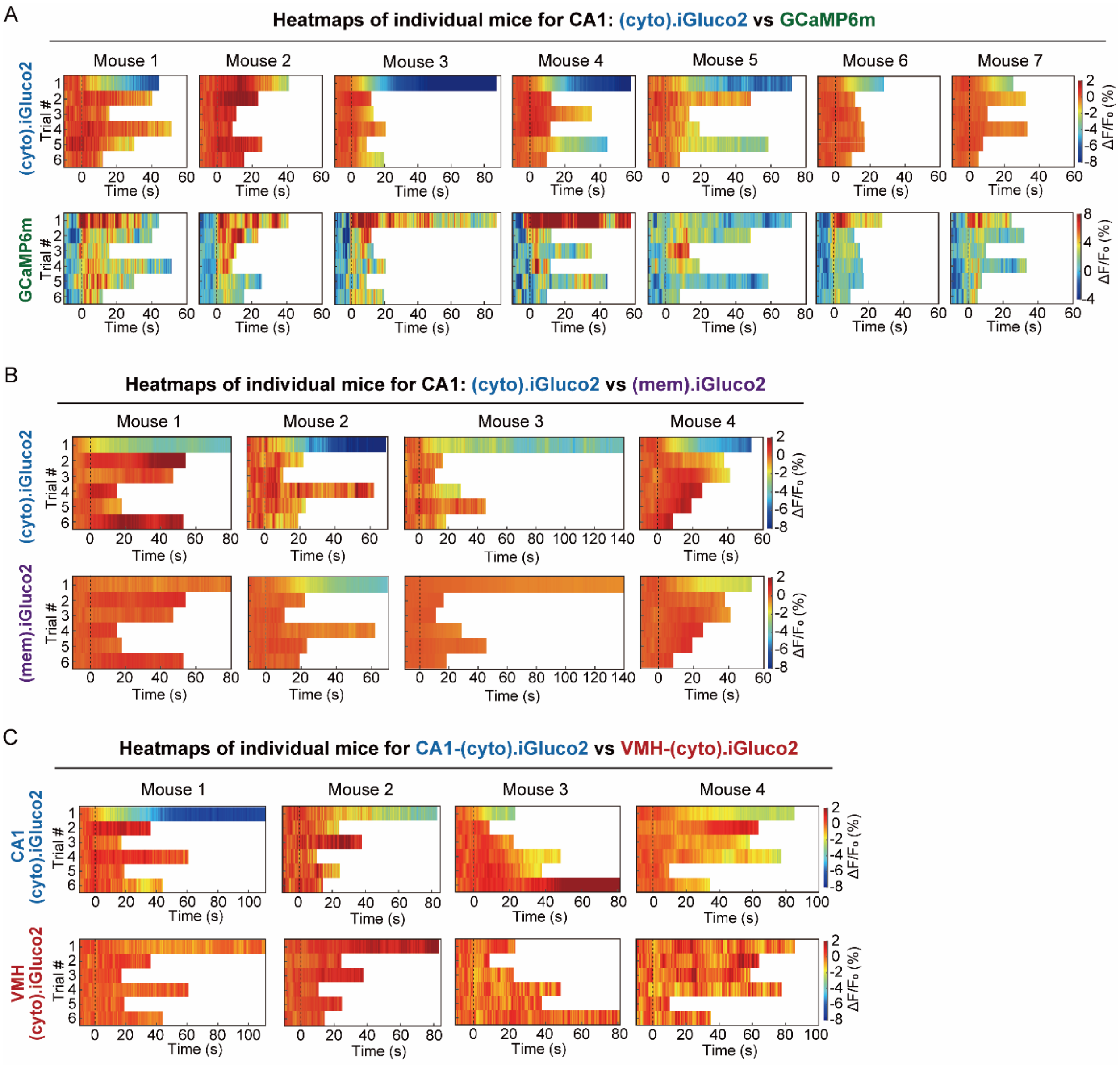

Fig. S3. Spatial navigation learning leads to a reduction in glucose consumption in CA1 neurons.

(A) Heatmaps from individual mice showing (cyto).iGluco2 and GCaMP6m signals in CA1 neurons across trials 1–6 during the maze task in Fig. 2 F-H.

(B) Heatmaps from individual mice showing (cyto).iGluco2 and (mem).iGluco2 signals in CA1 neurons across trials 1–6 during the maze task in Fig. 2 I-K.

(C) Heatmaps from individual mice showing (cyto).iGluco2 signals in CA1 and VMH neurons across trials 1–6 during the maze task in Fig. 2 L-N.

bioRxiv preprint doi: https://doi.org/10.64898/2026.07.01.735889; this version posted July 2, 2026. The copyright holder for this preprint (which was not certified by peer review) is the author/funder, who has granted bioRxiv a license to display the preprint in perpetuity. It is made available under a CC-BY 4.0 International license.

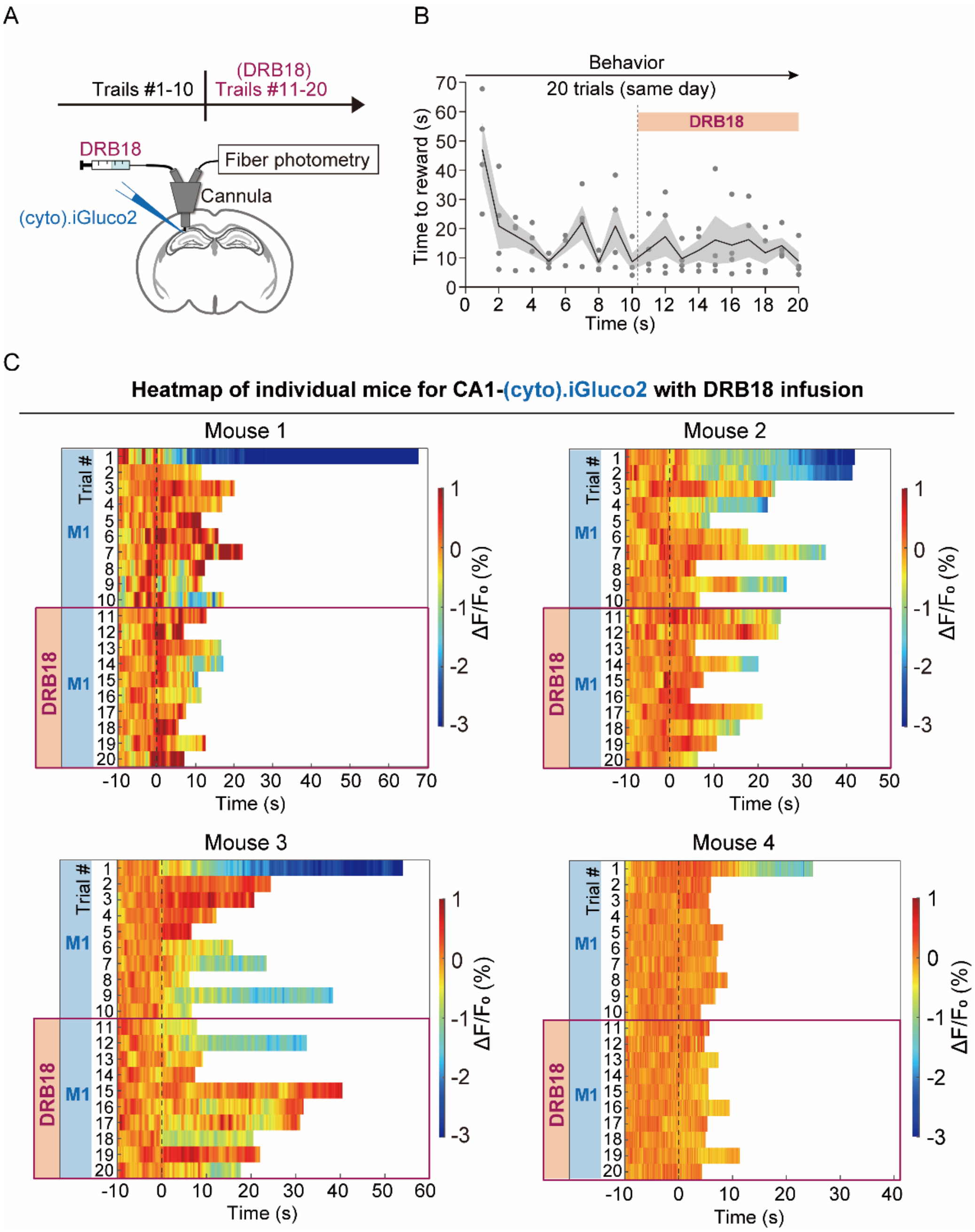

650 **Fig. S4. CA1 neurons maintain low glucose consumption in the familiar maze.**
651 (A) Schematic showing sensor injection and cannula placement for local drug infusion in CA1
652 during fiber photometry recording.
653 (B) Maze navigation learning curve showing the time to reward across trials 1–20 in the maze task
654 following DRB18 infusion (n = 4 mice).
655 (C) Heatmaps from individual mice showing (cyto).iGluco2 signals in CA1 neurons across trials
656 1–20 in the maze task following DRB18 infusion.

bioRxiv preprint doi: https://doi.org/10.64898/2026.07.01.735889; this version posted July 2, 2026. The copyright holder for this preprint (which was not certified by peer review) is the author/funder, who has granted bioRxiv a license to display the preprint in perpetuity. It is made available under a CC-BY 4.0 International license.

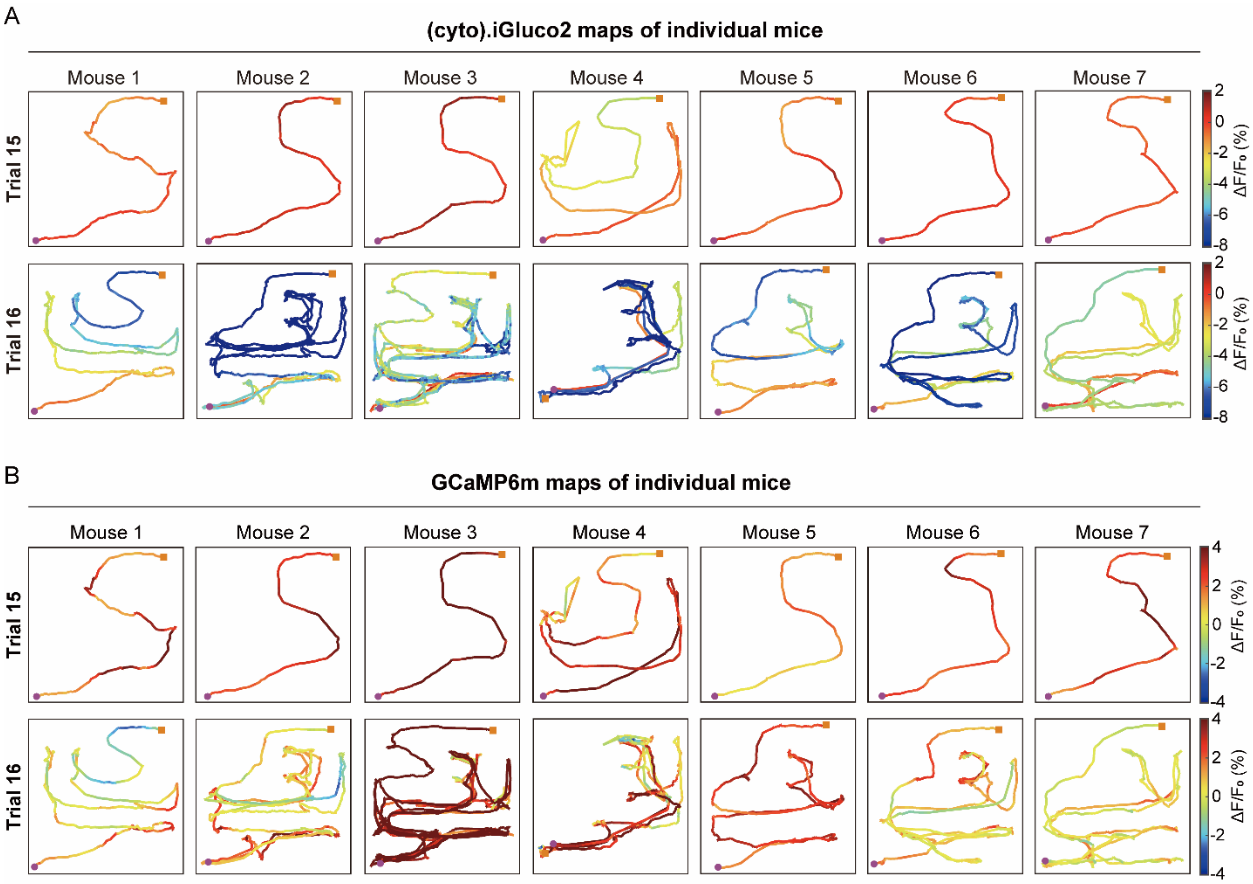

Fig. S5. Intracellular glucose and calcium dynamics in CA1 neurons during maze transition.
(A) Maps of (cyto).iGluco2 signal dynamics in CA1 neurons during trials 15 and 16 for individual mice.
(B) Maps of GCaMP6m signal dynamics in CA1 neurons during trials 15 and 16 for individual mice.

bioRxiv preprint doi: https://doi.org/10.64898/2026.07.01.735889; this version posted July 2, 2026. The copyright holder for this preprint (which was not certified by peer review) is the author/funder, who has granted bioRxiv a license to display the preprint in perpetuity. It is made available under a CC-BY 4.0 International license.

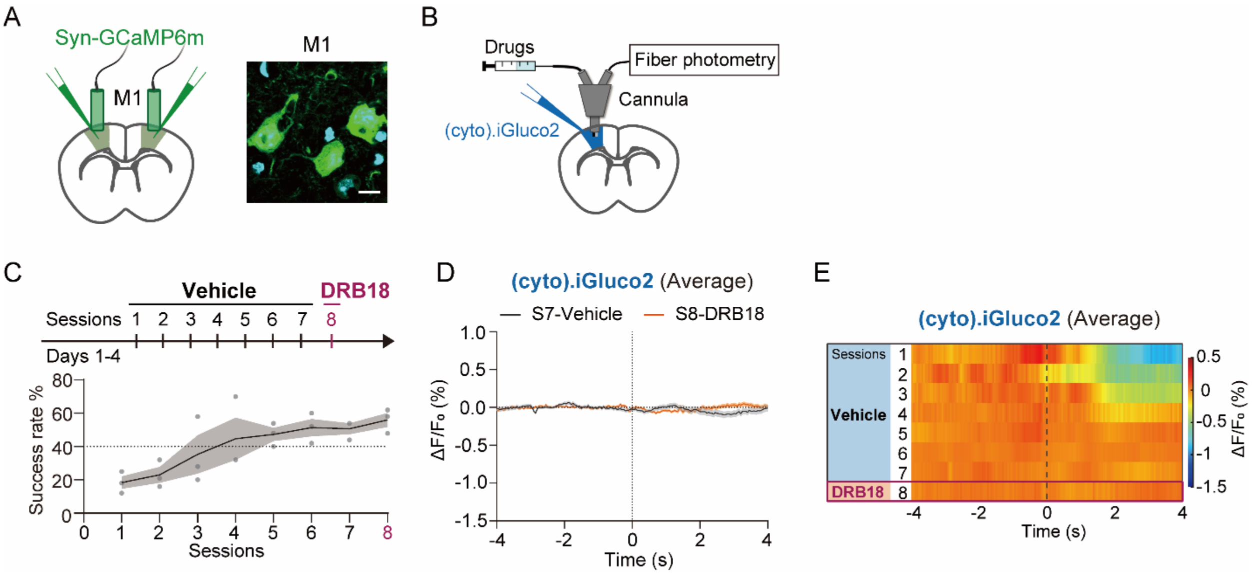

Fig. S6. M1 neurons maintain stably low glucose consumption after motor skill acquisition. (A) Schematic illustrating sensor injection and fiber placement in M1 cortex (left). Representative histology showing GCaMP6m expression in M1 neurons for fiber photometry recording (right, scale bar, 20 µm).
(B) Schematic showing sensor injection and cannula placement for local drug infusion in M1 during fiber photometry recording.
(C) Motor learning curve for M1 (cyto).iGluco2 mice showing success rate across sessions 1–8, with vehicle infused during sessions 1–7 and DRB18 infused in session 8 (n = 3 mice).
(D-E) Average (cyto).iGluco2 signal in M1 neurons aligned to the onset of successful reaches in session 7 and 8 (n = 3 mice).
